# Dynamic changes of serum SARS-Coronavirus IgG, pulmonary function and radiography in patients recovering from SARS after hospital discharge

**DOI:** 10.1186/1465-9921-6-5

**Published:** 2005-01-08

**Authors:** Lixin Xie, Youning Liu, Baoxing Fan, Yueyong Xiao, Qing Tian, Liangan Chen, Hong Zhao, Weijun Chen

**Affiliations:** 1 Department of Respiratory Medicine, Chinese PLA General Hospital, 28 Fuxing Road, Beijing, 100853, P.R. China; 2 Department of Radiology, Chinese PLA General Hospital, 28 Fuxing Road, Beijing, 100853, P.R. China; 3 BGI-GBI Biotech Company, Beijing, P.R. China

**Keywords:** Severe acute respiratory syndrome (SARS), SARS-CoV IgG antibody, Pulmonary function, Pulmonary fibrosis, Avascular necrosis of femoral head

## Abstract

**Objective:**

The intent of this study was to examine the recovery of individuals who had been hospitalized for severe acute respiratory syndrome (SARS) in the year following their discharge from the hospital. Parameters studied included serum levels of SARS coronavirus (SARS-CoV) IgG antibody, tests of lung function, and imaging data to evaluate changes in lung fibrosis. In addition, we explored the incidence of femoral head necrosis in some of the individuals recovering from SARS.

**Methods:**

The subjects of this study were 383 clinically diagnosed SARS patients in Beijing, China. They were tested regularly for serum levels of SARS-CoV IgG antibody and lung function and were given chest X-rays and/or high resolution computerized tomography (HRCT) examinations at the Chinese PLA General Hospital during the 12 months that followed their release from the hospital. Those individuals who were found to have lung diffusion abnormities (transfer coefficient for carbon monoxide [D_L_CO] < 80% of predicted value [pred]) received regular lung function tests and HRCT examinations in the follow-up phase in order to document the changes in their lung condition. Some patients who complained of joint pain were given magnetic resonance imaging (MRI) examinations of their femoral heads.

**Findings:**

Of all the subjects, 81.2% (311 of 383 patients) tested positive for serum SARS-CoV IgG. Of those testing positive, 27.3% (85 of 311 patients) were suffering from lung diffusion abnormities (D_L_CO < 80% pred) and 21.5% (67 of 311 patients) exhibited lung fibrotic changes. In the 12 month duration of this study, all of the 40 patients with lung diffusion abnormities who were examined exhibited some improvement of lung function and fibrosis detected by radiography. Of the individuals receiving MRI examinations, 23.1% (18 of 78 patients) showed signs of femoral head necrosis.

**Interpretation:**

The lack of sero-positive SARS-CoV in some individuals suggests that there may have been some misdiagnosed cases among the subjects included in this study. Of those testing positive, the serum levels of SARS-CoV IgG antibody decreased significantly during the 12 months after hospital discharge. Additionally, we found that the individuals who had lung fibrosis showed some spontaneous recovery. Finally, some of the subjects developed femoral head necrosis.

## Introduction

Severe acute respiratory syndrome (SARS) is a new infectious disease in humans. The first victim of SARS to be diagnosed was a businessman from the city of Foshan in Guangdong Province, China. SARS patients may present with a spectrum of symptoms and signs, ranging from relatively asymptomatic to fulminant pneumonitis and death [1]. Lung injury caused by the SARS coronavirus (SARS-CoV) is one of the main clinical manifestations in SARS patients, significantly affecting their prognosis. A regular follow-up survey of SARS patients in the convalescent phase would be helpful to evaluate any changes in acquired immune function, pulmonary function, bones and joints over the course of time. At present, there have been few reports about the relationship between the prognosis for recovery and the degree of lung injury caused by the SARS-CoV. In addition, a study of the serum levels of the specific IgG antibody against SARS-CoV is needed because it is the major immunologic protection to aid in recovery and is essential to avoid repeated infection with SARS-CoV. It has been 14 months since the World Health Organization officially declared the global outbreak of SARS to be under control [2]. The present study focused on the dynamic changes in the IgG antibody levels against SARS-CoV and in lung lesions in the discharged but recovering SARS patients as measured by lung function and imaging tests. The phenomenon of femoral head necrosis was also investigated in those SARS patients who complained of chronic bone and joint pain during the one year follow-up after discharge from the hospital.

## Methods

All of the subjects of this study were discharged from Beijing Xiaotangshan Hospital, Beijing Armed Police Hospital, and Chinese 309 PLA Hospital, and all gave their informed consent.

### Study Protocol

The subjects of our investigation were 383 clinically diagnosed SARS patients in the convalescent phase (160 male and 223 female, average age 38.2 ± 13.6 years) undergoing testing from May, 2003 to June, 2004. Each clinical diagnosis was based on the Clinical Diagnosis Standard for SARS Patients issued by the Ministry of Chinese Public Health [3]. All participants in the study had met the specified criteria for discharge from the hospital [4]. On the first visit, each patient was given a routine pulmonary function test (ventilation and diffusion function: SensorMedics 2200 pulmonary function test apparatus, U.S.A.), a chest X-ray examination and serum SARS-CoV specific antibody (SARS-CoV IgG) test at the Chinese PLA General Hospital, Beijing, P.R. China. Those individuals suspected of having pulmonary fibrotic changes received high resolution computerized tomography (HRCT) examination of their lungs. Individuals who complained of chronic pain in their bones and joints or who had difficulty walking received femoral head magnetic resonance imaging (MRI) examinations.

Each patient returned a month after the first visit followed by one visit every 3 months. Serum SARS-CoV IgG was tested at each return visit. If negative results were obtained twice consecutively, the case was regarded as a misdiagnosis and the patient did not undergo a follow-up survey. Patients with positive SARS-CoV IgG and abnormal pulmonary diffusion received regular pulmonary function tests and those showing pulmonary fibrosis in imaging examinations received further regular HRCT examinations. Some individuals observed to have avascular necrosis of the femoral head received MRI examinations 3–6 months later.

### Clinical Diagnostic Criteria for the Patients with SARS Disease in Mainland China [3]

#### (1) Epidemiological history

(1.1) The individual has a history of close contact with SARS patients or is part of a cluster of cases of SARS or there is clinical evidence of having infected other patients.

(1.2) The individual has a history of recent travel to an area where SARS cases have been reported within 2 weeks and secondary infected SARS cases have been found.

#### (2) Symptoms and signs

Acute onset of SARS generally begins with a prodrome of fever with a temperature >38°C, sometimes accompanied by chills, myalgia and anthralgia, headaches, and fatigue. Upper respiratory tract symptoms of catarrh are not prominent, although cough may be present. If present, it is mainly a dry cough, occasionally with blood streak sputum. Some individuals have chest discomfort, and severe cases may present with tachypnea, panting, and even respiratory distress.

Generally, there are no obvious pulmonary signs among SARS patients. Wet rales and signs of lung consolidation, as well as decreased respiration and other signs of pleural effusion can occasionally be found.

Note: Some patients do not show initial symptoms of fever, especially those who have had recent surgery or those having chronic diseases.

#### (3) Routine laboratory examinations

White blood cell counts are generally normal or below normal, with decreased absolute lymphocyte counts.

#### (4) Chest radiological examinations

The typical imaging profile of SARS is of multiple patchy opacities with bilateral distribution. The opacities are usually ground-glass in appearance, sometimes with air space consolidation, evolving progressively over the course of the disease. The evolution is very rapid in some cases, resulting in the confluence of lesions and large areas of opacification in a short time. If the chest radiological examination is negative, reexamination after 1 to 2 days should be done.

#### (5) Antibiotic therapy is ineffective

***Suspected cases: ***In accordance with 1.1+2+3, 1.2+2+4 or 2+3+4.

***Clinically diagnosed cases: ***In accordance with 1.1+2+4, 1.2+2+4+5, or 1.2+2+3+4.

### SARS-CoV IgG Antibody Test

The SARS-CoV IgG antibody in serum specimens from recovering SARS patients was assayed by the BGI-GBI Biotech Company with an enzyme-linked immunosorbent assay (ELISA) kit (No. S20030003, BGI-GBI Biotech Company, Beijing, P.R. China). The wells containing polystyrene microplate strips were coated with two recombinant SARS-CoV antigens that are well-characterized. Recovering SARS patients' serum samples in the diluent buffer (1:10) were incubated in the coated wells for 30 min at 37°C and then the wells were washed 5 times with the washing buffer. The dilutedenzyme-labeled anti-human IgG (100 μl) was added to the wells and incubated for 20 min at 37°C. The wells were washed 5 times with the washing buffer. A tetramethyl-benzidine substrate was then added to each well. The presence of specific antibodies was indicated by a yellow color developing after substrate addition. The reaction was terminated by addition of hydrochloric acid. The intensity of the color was measured spectrophotometrically at 450 nm to quantify the amount of antibody in the specimen. The optical density (OD) measured was compared with a standard calibration curve constructed for each lot, yielding concentration values for the samples. The OD values of both the positive and negative controls were determined. The threshold value for IgG was 0.18 OD units, calculated as the mean + 2 standard deviation (SD) levels of the readings given by 1000 control blood donor sera samples. If the OD was above the threshold value, the sample was considered to be positive for SARS-CoV IgG [5].

### Pulmonary Function Test

Each recovering SARS patient underwent a standard pulmonary function test (SensorMedics 2200, Yorba Linda, U.S.A) for forced expiratory volume in 1 second (FEV_1_), vital capacity (VC), forced vital capacity (FVC), total lung capacity (TLC), transfer coefficient for carbon monoxide (D_L_CO), and carbon monoxide diffusion constant (D_L_CO/V_A_) measured by means of the single-breath test. The hemoglobin level was also measured to adjust the D_L_CO value. The results were compared with those of age- and sex-matched controls and expressed as a percentage of predicted values. Pulmonary function was regarded as abnormal when the D_L_CO was less than 80% of predicted values (pred). This was considered a diffusion deficit [6].

### Chest Radiography and Evaluation

Frontal chest X-ray radiographs (CXR) were obtained at the first follow-up visit for each recovering SARS patient. If abnormities were found in the CXR or if the D_L_CO was <80% pred despite a normal CXR, the patient was sent for HRCT scanning (GE Light Speed, GE, U.S.A. 1-mm section in thickness with a 10-mm gap, supine position, scanning during inspiration, 1 second per scan, 140 kv, 200 mA). All CXR and HRCT images were assessed by three radiologists via a viewing console. The three radiologists were aware of the patients' clinical diagnosis at the time of their review of the radiographs. The final conclusions were established by consensus. Each segment of the lung was reviewed for ground-glass opacification, interstitial thickening, bronchiectasis, and architectural distortion. Abnormalities were magnified by means of a zoom function and were examined for intralobular interstitial, interlobular septal, or peribronchovascular interstitial thickening. Attention was also paid to the presence or absence of nodules or masses, cavitation or calcification, and emphysema. The presence of parenchymal bands, irregular interfaces (bronchovascular, pleural, or mediastinal), thickened interstitium, and traction bronchiectasis were considered as evidence of fibrotic changes [7].

### Magnetic Resonance Imaging (MRI) Examination

All MRI examinations were done using a 1.5 T Signa CVi imager (GE Medical Systems, Milwaukee, WI, U.S.A.). For the patients who complained of chronic bone and joint pain, coronal T_1_-weighted (spin echo; time to repetition [TR], 440–500; time to echo [TE) 11–14] scans of the hips were done. If there were any abnormalities noted in the T_1_-weighted images, further T_1_-weighted sagittal images and coronal short tau inversion recovery (inversion time 145, TR 3500–5000, TE 80–120) or turbo-spin-echo T_2_-weighted images with fat suppression (TR 2500–3000, TE 80–120) were obtained. Images 3 mm thick were obtained for the coronal studies, and 4 mm thick images were obtained for the sagittal studies. Osteonecrosis was diagnosed by the presence of a band of low signal intensity in T_1_-weighted images [8].

### Statistical Analysis

All data were expressed as the  ± SD unless otherwise indicated. Statistical analyses were done by one-way analysis of variance (ANOVA), Student-Newman-Keuls, and Chi-square test for multiple comparisons. We used the STATA™ 7.0 statistical analysis software for Windows (STATA Statistical Software, Inc., U.S.A.) for evaluating the results of our study. With each statistical test, the criterion for significance was a *p *value of less than 0.05.

## Results

The interval from hospital discharge to the first follow-up visit was 45.0 ± 20.7 days (Range: 11–104 days). Of the 383 individuals participating in our study, 311 patients (81.2%) tested positive for SARS-CoV IgG and 72 (18.8%) tested negative. (All patientswere tested twice for SARS-CoV IgG.) Of these, 33 patients (13 male and 20 female, average age 35.7 ± 12.1) with positive SARS-CoV IgG and abnormal pulmonary diffusion received regular follow-up examinations each month, from June to December in 2003, and every two months, from January to June in 2004. Tables 1 and 2 show the dynamic changes of SARS-CoV IgG in patients with positive tests for SARS-CoV IgG within the year after discharge, indicating that the serum SARS-CoV IgG remained at high levels, although it decreased significantly over the course of time.

**Table 1:** Dynamic changes of serum SARS-CoV IgG antibody levels in patients recovering from SARS

	Samples (n)	± SD (OD units)
May, 2003	35	1.240 ± 0.350
June, 2003	74	1.087 ± 0.284
July, 2003	172	1.203 ± 0.306
Aug., 2003	152	1.061 ± 0.376
Sept., 2003	123	1.105 ± 0.378
Oct., 2003	35	1.097 ± 0.282
Nov., 2003	77	0.835 ± 0.327†‡§¶*
Dec., 2003	35	0.829 ± 0.232†§*
Jan.–Feb., 2004	67	0.737 ± 0.169†‡§¶*#
Mar–Apr, 2004	34	0.678 ± 0.179†‡§¶*#
May–June, 2004	46	0.621 ± 0.181†‡§¶*#
F value		30.62
*p *value		0.0000

Note: Statistical analyses were done by one-way analysis of variance (ANOVA) and Student-Newman-Keuls for multiple comparisons, and values are given as mean ± SD;

† *p *< 0.05 vs. SARS-CoV IgG antibody results in May, 2003.

‡ *p *< 0.05 vs. SARS-CoV IgG antibody results in June, 2003.

§ *p *< 0.05 vs. SARS-CoV IgG antibody results in July, 2003.

¶ *p *< 0.05 vs. SARS-CoV IgG antibody results in August, 2003.

* *p *< 0.05 vs. SARS-CoV IgG antibody results in Sepember, 2003.

# *p *< 0.05 vs. SARS-CoV IgG antibody results in October, 2003.

**Table 2:** Dynamic changes of serum SARS-CoV IgG antibody levels in 33 regular follow-up examinations of patients recovering from SARS

	Samples (n)	± SD (OD units)
June, 2003	33	1.104 ± 0.267
July, 2003	33	1.325 ± 0.357
Aug., 2003	33	1.092 ± 0.249
Sept., 2003	33	1.121 ± 0.432
Oct., 2003	33	1.056 ± 0.309
Nov., 2003	33	0.895 ± 0.203‡¶
Dec., 2003	33	0.800 ± 0.170†‡§¶
Jan.–Feb., 2004	33	0.726 ± 0.163†‡§¶*
Mar–Apr, 2004	33	0.675 ± 0.181†‡§¶*
May–June, 2004	33	0.610 ± 0.167†‡§¶*#
*F *value		25.69
*p *value		0.0000

Note: Statistical analyses were done by one-way analysis of variance (ANOVA) and Student-Newman-Keuls for multiple comparisons, and values are given as mean ± SD;

† *p *< 0.05 vs. SARS-CoV IgG antibody results in June, 2003.

‡ *p *< 0.05 vs. SARS-CoV IgG antibody results in July, 2003.

§ *p *< 0.05 vs. SARS-CoV IgG antibody results in August, 2003.

¶ *p *< 0.05 vs. SARS-CoV IgG antibody results in September, 2003.

* *p *< 0.05 vs. SARS-CoV IgG antibody results in October, 2003.

# *p *< 0.05 vs. SARS-CoV IgG antibody results in November, 2003.

There were 88 individuals (23.0%) with abnormal D_L_CO among the 383 patients participating in our study. Of the 311 individuals testing positive for SARS-CoV IgG, there were 85 with abnormal D_L_CO (27.3%, 85/311), in contrast to just 3 cases with abnormal D_L_CO among the 72 subjects testing negative for SARS-CoV IgG (4.2%, 3/72). There was a statistically significant difference between positive and negative SARS-CoV IgG groups in D_L_CO values (table 3).

Among the 85 patients (29 male and 56 female, average age 42.2 ± 11.9 years) with abnormal D_L_CO and positive SARS-CoV IgG, 40 individuals received pulmonary function tests 4 times within the year at 42.0 ± 10.4, 70.0 ± 11.8 and 155.1 ± 42.9 day intervals. Among these 40 patients, there were 23 who exhibited abnormal D_L_CO at their second pulmonary function examination, 23 at the third examination, and 20 at the fourth examination (table 4).

.

**Figure 1:**
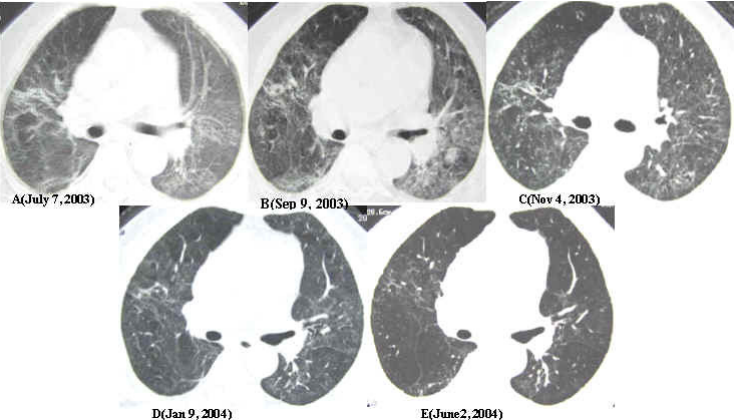
The results of chest HRCT examination in a SARS patient in the convalescent phase, showing marked reversal of pulmonary fibrosis.

Pulmonary fibrosis was detected by CXR and confirmed by HRCT examination in 72 SARS patients in the convalescent phase. Among these, there were 4 individuals with negative and 67 with positive SARS-CoV IgG. Of the 40 patients who received HRCT examinations at least 4 times, all showed improvement in the fibrotic condition (Figure 1).

Of the 311 convalescent SARS patients with sero-positive SARS-CoV IgG, 78 received femoral head MRI examinations. The Imaging showed that 18 of these patients (23.1%, 18/78) had avascular necrosis of the femoral head. Of these 18 individuals, 8 had avascular necrosis of both femoral heads and 10 had avascular necrosis of one femoral head. Ten of the 18 patients showed first stage changes and 8 had secondary changes. During the 3–6 month follow-up visits for these individuals, there were no obvious changes in the avascular necrosis for these patient.

## Discussion

Since the outbreak of SARS at the end of 2002, despite the great efforts that have been extended, the mechanisms, clinical characteristics, prognosis and effective therapeutics for this disease have not been adequately clarified. Both the SARS virus itself and the anti-viral therapy (such as high-dose glucocorticoids) used in treatment can cause various degrees of toxicity and side effects, including pulmonary fibrosis and avascular necrosis of the femoral head, even in the convalescent phase. Follow-up surveys of SARS patients in the convalescent phase are needed for recognizing the clinical characteristics of this disease and reevaluation of the therapeutic treatments [2,7].

In our study, 72 individuals (18.8%) showed negative results in the SARS-CoV IgG antibody test for at least two tests, suggesting that there may have been misdiagnosis of some clinically diagnosed SARS patients. Comparison of the Chinese clinical diagnosis standard (published April, 2003) [3] to the Center for Disease Control (CDC) SARS case definition (published April 30, 2003) [9], indicates that both of them emphasize the importance of epidemiological history, clinical manifestations and chest radiological changes for the clinical diagnosis of SARS disease. The CDC SARS case definition especially emphasizes the importance of laboratory criteria for the confirmation of a SARS diagnosis. This is accomplished by detecting the dynamic changes in the titration of specific antibodies against SARS CoV and positive detection of SARS-CoV RNA by PCR. In contrast, the Chinese clinical diagnosis standard did not mention the importance of a laboratory SARS-CoV test for the confirmation of a SARS diagnosis. This might have resulted in the misdiagnosis of SARS in some cases. During follow-up examinations, we found that those individuals with positive SARS-CoV IgG remained positive for a year, although the level of the antibody decreased gradually. Therefore, those inoculated with a SARS vaccine or infected by the SARS virus might not receive lifetime immunity, but only immunity for a limited duration. Certainly, our findings must be confirmed by further studies [7,8].

By regular examination of pulmonary function and CXR, we found that those with pulmonary fibrotic changes were able to heal on their own. The fibrotic tissue was absorbed and pulmonary diffusion and VC improved with time, suggesting that the mechanism of lung injury and lung fibrosis caused by the SARS-CoV may have a different pathophysiological process compared to other lung diseases, such as idiopathic pulmonary fibrosis or pulmonary fibrosis secondary to adult respiratory stress syndrome. The reason is not clear. However, in our follow-up study, we found some ground-glass-like changes in the HRCT images from SARS patients one year after discharge. This result shows that changes in the lung can still be observed in convalescents [7,9].

Recent concern has focused on a complication of SARS in the convalescent phase, when avascular necrosis develops on the femoral head. The morbidity of this condition is reported to be 15% to 30% in some SARS patients in Mainland China [8,10]. Among the 78 patients receiving an MRI examination, there were 18 cases of complicated necrosis of the femoral head to different degrees. The causes of this complication include SARS itself and the drugs (such as glucocorticoids) used in treatment, with the latter being more important than the former [11-13]. We didn't find any worsening or improvement of the avascular necrosis of the femoral head in these patients during our follow-up examinations. Although most patients received magnetotherapy, hyperbaric oxygen chamber therapy, local kerotherapy and Chinese traditional medicine to promote local blood circulation, there was no apparent short-term therapeutic effectiveness of these methods for recovery of the femoral head.

In conclusion, SARS, as a new disease, remains unfamiliar to mankind. It has high rates of morbidity and mortality in the acute phase. A significant proportion of patients surviving the acute illness have impairment in their overall functional capacity and health status in the convalescent phase after discharge from the hospital. Follow-up surveys of SARS patients in the convalescent phase are needed to understand the clinical characteristics of this disease. Our findings suggest that follow up studies of these patients are required for a longer duration, including comprehensive assessments for detection and appropriate management of any persistent or emerging sequelae. These types of investigation may facilitate the search for effective therapeutics and aid in ultimately conquering this disease.

**Table 3:** D_L_CO results for the convalescent SARS patients with sero-positive or sero-negative SARS-CoV IgG

	Positive	Negative	Total	X^2^	*P *value
D_L_CO normal cases	226	69	295		
D_L_CO abnormal cases	85	3	88		
Total	311	72	383	17.7269	0.000

Note: Analyzed with Chi-square test.

**Table 4:** Pulmonary function test results from the 4 follow-up examinations of 40 convalescent SARS patients ( ± SD)

Follow-up*	VC(% pred)	FEV_1_(% pred)	D_L_CO(% pred)	D_L_CO/V_A_(% pred)
Two months	87 ± 15 (51~114)	83 ± 13 (60~108)	69 ± 9 (47~79)	95 ± 14 (58~123)
Four months	94 ± 14 (61~123)	90 ± 13 (65~121)	76 ± 11 (48~94)	99 ± 14 (67~126)
Six months	100 ± 15† (66~136)	93 ± 12† (66~114)	76 ± 11† (52~98)	97 ± 14 (62~129)
Eleven months	103 ± 15† (66~142)	96 ± 11† (67~115)	79 ± 12† (56~98)	97 ± 14 (59~128)
*F *value	9.23	7.84	6.15	0.63
*P *value	0.0000	0.0001	0.0006	0.5936

Note: *: Indicating as time after discharge from acute illness.

Statistical analyses were done by one-way analysis of variance (ANOVA) and Student-Newman-Keuls for multiple comparisons, and values are given as mean ± SD;

†: Compared to those in the first follow-up exam, *p *< 0.05.
